# Conditioned medium of human adipose-derived mesenchymal stem cells mediates protection in neurons following glutamate excitotoxicity by regulating energy metabolism and GAP-43 expression

**DOI:** 10.1007/s11011-014-9490-y

**Published:** 2014-01-25

**Authors:** Peng Hao, Zhanhua Liang, Hua Piao, Xiaofei Ji, Yachen Wang, Yong Liu, Rutao Liu, Jing Liu

**Affiliations:** 1 Regenerative Medicine Centre, First Affiliated Hospital of Dalian Medical University, No.222 Zhongshan Road, Dalian, 116011 People’s Republic of China; 2 Institute of Integrative Medicine, Dalian Medical University, Dalian, 116044 People’s Republic of China; 3 Department of Neuroscience, First Affiliated Hospital of Dalian Medical University, Dalian, 116011 People’s Republic of China; 4 College of Basic Medical Sciences, Dalian Medical University, Dalian, 116044 People’s Republic of China; 5 Department of Traditional Chinese Medicine, First Affiliated Hospital of Dalian Medical University, Dalian, 116011 People’s Republic of China; 6 Department of Neurobiology, School of Basic Medical Sciences, Capital Medical University, Beijing, 100069 People’s Republic of China

**Keywords:** Excitotoxicity, Cortical neurons, AMSCs, Neuroprotection, GAP-43, Energy metabolism

## Abstract

Glutamate excitotoxicity has been implicated as one of the pathological mechanisms contributing to neuronal cell death and is involved in many neurological disorders. Stem cell transplantation is a promising approach for the treatment of nervous system damage or diseases. Previous studies have shown that mesenchymal stem cells (MSCs) have important therapeutic effects in experimental animal and preclinical disease model of central nervous system pathology. However, it is not well understood whether neurogenesis of MSCs or MSC conditioned-medium (CM) containing microparticles mediates therapeutic effects. Here, we investigated the neuroprotective effects of human adipose-derived MSCs (AMSCs) on cortical neurons using models of glutamate excitotoxicity. Following exposure to glutamate (100 μM, 15 min), cortical neurons were co-cultured with either AMSCs separated by a semiporous membrane (prohibiting direct cell-cell contact) or with AMSC-CM for 18 h. Compared to untreated control groups, AMSCs and AMSC-CM partially and similarly reduced neuronal cell damages, as indicated by reduced LDH release, a decreased number of trypan-positive cells and a decline in the number of apoptotic nuclei. Protection by CM was associated with increased GAP-43 expression and an elevated number of GAP-43-positive neurites. Furthermore, CM increased levels of ATP, NAD^+^ and NADH and the ratio of NAD^+^/NADH, while preventing a glutamate-induced decline in mitochondrial membrane potential. These results demonstrate that AMSC-CM mediates direct neuroprotection by inhibiting neuronal cell damage/apoptosis, promoting nerve regeneration and repair, and restoring bioenergy following energy depletion caused by glutamate excitotoxicity.

## Introduction

Excitotoxicity is defined as cell death resulting from the toxic action of excitatory amino acids. Glutamate is the major excitatory neurotransmitter in the mammalian central nervous system (CNS) and it is involved in many physiological functions including development of the nervous system, learning and memory. However, excessive and prolonged exposure to glutamate under pathological circumstances can lead to overstimulation of glutamate receptors and excitotoxic cell death (Lau and Tymianski 2010). Glutamate excitotoxicity is implicated as a common pathological mechanism contributing to neuronal cell injury in a number of neurological disorders as it plays a critical role in the pathogenesis of stroke, epilepsy, brain trauma and neurodegenerative diseases (Lipton and Rosenberg 1994; Dong et al. 2009).

It is clear that the glutamate-induced neuronal death is mediated by the entry of extracellular Ca^2+^ as a result of the activation of N-Methyl-D-aspartic acid (NMDA) subtype of glutamate receptors and a resultant calcium overload (Arundine and Tymianski 2003). Excessive intracellular calcium triggers pathways leading to cell death. Indeed, it has been suggested that intracellular calcium overload may result in mitochondrial dysfunction, energy metabolism disorder, oxidative stress and apoptosis. Recent studies have suggested that mitochondrial dysfunction and subsequent energetic collapse are critical steps in the progression to cell death in glutamate excitotoxicity (Nicholls 2004; Smaili et al. 2011).

Bioenergy homeostasis is foundational for maintaining normal cell function and survival. Neurons are excitable cells that require a large amount of energy to maintain membrane potential and synaptic transmission, making the brain the most energy -consuming organ in the body. Thus, neurons are extremely susceptible to bioenergetic stress. Irreversible neuronal death occurs if the brain is deprived of oxygen for more than 5 min. Among many neural activities, the excitatory glutamatergic systems use the most energy (Sibson et al. 1998; Shen et al. 1999; Raichle and Gusnard 2002). The energy consumption of neurons increases following activation by glutamate. Energy metabolism disorder is involved in multiple acute and chronic neurological diseases including Alzheimer’s disease, Parkinson’s disease, Huntington’s disease, stroke and brain trauma (Mattson et al. 1999; Ferreira et al. 2010; Amato and Man 2011; Mochel et al. 2012). Reduced cell energy supply is one of the early and primary pathological events in these diseases. Furthermore, under these pathological conditions, neural energy depletion is accompanied by a massive release of glutamate and excitotoxic cell injury (Del Rio et al. 2007; Nicholls et al. 2007). Hence, minimizing glutamate-induced neuronal cell injury and rescuing glutamate-induced neuronal energy depletion is a direct and effective therapeutic approach.

One repair strategy following nerve injury is to promote axonal regeneration or outgrowth. In recent years, several molecules have been found associated with axonal regeneration, and among them the most well-known is the growth-associated protein 43 (GAP-43) (Frey et al. 2000). GAP-43, which was discovered in the early 1980s, is a membrane-bound protein expressed mainly in the growth cores, axons and presynaptic terminals of neurons (Benowitz et al. 1987). It is particularly abundant in periods of neurite outgrowth during development and regeneration in the central and peripheral nervous systems. GAP-43 is closely related to neural development, axonal regeneration and synaptic reorganization, and is regarded as an intrinsic factor during development and regeneration (Benowitz and Routtenberg 1997). Therefore, GAP-43 is considered as an axonal regeneration marker (Aoki et al. 2007) and a neural restoration marker (Lopatina et al. 2011).

As the capacity of the CNS to regenerate after acute or chronic lesions is limited, there are currently no effective therapeutic strategies for many neurological diseases. Recently, however, many studies indicate that stem cell transplantation is a promising approach for the treatment of nervous system damages or diseases. Somatic stem cells, especially neural stem cells and mesenchymal stem cells (MSCs), have demonstrated the character to reduce neurological defects and promote functional recovery in experimental animal models of CNS pathology (Hofstetter et al. 2002; Zhang et al. 2006; Ohtaki et al. 2008) and in preclinical trials for stroke and multiple sclerosis (Bang et al. 2005; Freedman et al. 2010; Martino et al. 2010).

The mechanisms by which MSCs provide neuroprotection under CNS pathological conditions are not well known. Early research showed that MSCs could differentiate into CNS glial cells and neurons in vitro and in vivo (Kopen et al. 1999; Sanchez-Ramos 2002). But it is controversial whether MSCs are able to functionally replace damaged neural cells besides expressed neuronal or glial markers (Phinney and Prockop 2007). There is evidence that MSCs stimulate endogenous protective and restorative responses via paracrine mechanisms (Uccelli et al. 2008). Recent studies on MSCs secretome suggest that MSCs secrete numerous bioactive factors that mediate neuroprotection via trophic support, immunomodulation, anti-apoptosis, anti-fibrosis and angiogenesis (Singer and Caplan 2011; Skalnikova et al. 2011). MSCs were found to protect against glutamate-induced apoptosis in rat pheochromocytoma cell line PC12 cells through the secretion of neurotrophic factors (Lu et al. 2011). Growth factors such as BDNF and bFGF protected cerebellar granular neurons against glutamate excitotoxicity by increasing glucose reuptake and stabilizing intracellular Ca^2+^ and the mitochondrial electrochemical gradient (El Idrissi and Trenkner 1999). IGF-1 was demonstrated to reverse a decrease in GAP-43 expression induced by glutamate in dorsal root ganglion neurons (Liu et al. 2012). Adult adipose-derived MSCs (AMSCs) are particularly attractive for use in cell transplantation as they can be obtained and expanded in vitro with a relative ease. However, it is still unknown whether neurogenesis of MSCs or MSC-CM containing rich microparticles can prevent glutamate-induced energy depletion in neurons.

In the present study, in vitro models of glutamate excitotoxicity were used to investigate whether AMSC-CM mediate neuroprotection in cultured cortical neurons by regulating GAP-43 expression and energy metabolism, in order to provide an experimental basis for further exploration of AMSCs use for clinical applications.

## Experimental procedures

### Cell culture

#### Culture and identification of human AMSCs

Human adipose tissue (lipoaspirate) from healthy 35 to 45 year-old donors was obtained by liposuction procedures under anesthesia. All donors were informed and gave their written consent with procedures approved by the Ethics Committee on the Use of Human Subjects (Dalian Medical University and affiliated hospitals). AMSCs were isolated as previously described (Zuk et al. 2002). In brief, adipose tissue was washed in phosphate-buffered saline (PBS) and digested with 0.1 % collagenase type І (sigma) in PBS for 30 min at 37 °C in a water-bath shaker. The digested tissue was centrifuged for 5 min at 1,000 rpm at room temperature and the supernatant containing mature adipocytes, debris and connective tissue was aspirated. The pellet was resuspended and plated in T25 culture flasks in Dulbecco’s modified Eagle medium (DMEM; supplemented with D-glucose 4,500 mg/l, 4 mM L-glutamine and 110 mg/l sodium pyruvate; GIBCO, Invitrogen), 10 % fetal bovine serum (FBS; GIBCO, Invitrogen) and 100 U penicillin/100 μg streptomycin (GIBCO, Invitrogen) at a density of 2 × 10^6^/cm^2^. Cultures were maintained at 37 °C in a humidified atmosphere of 95 % O_2_ and 5 % CO_2._ After reaching 80–100 % confluence, cells were passaged using 0.25 % trypsin/0.38 % EDTA (GIBCO, Invitrogen) and kept in medium as described above. All experiments were performed with cells passages 4–6.

AMSCs were routinely characterized by flow cytometry analysis using antibodies to the surface markers CD13, CD34, CD106 (PE-conjugated mouse anti-human; BD Biosciences) and CD44, CD45, CD90 (FITC-conjugated mouse anti-human; BD Biosciences). AMSCs were positive (>95 %) for CD13, CD44 and CD90 and negative (<5 %) for CD34, CD45 and CD106, indicating their mesenchymal nature. AMSCs differentiation into adipocytes and osteoblasts was performed as follows. AMSCs were seeded at a density of 10^5^/ml in 35 mm dishes and grown in DMEM growth medium. After reaching 80–100 % confluence, mediums were replaced with adipocyte or osteoblast induction mediums (Hyclone). After 14 days of differentiation, adipocytes were stained with Oil Red O (Sigma) and fresh red lipid vacuoles were observed in the cytoplasm of nearly all AMSCs. After 28 days of osteoblast differentiation, cells were stained with Von Kossa and showed the mineralized matrix deposition.

#### Primary neuron culture

All protocols were performed in accordance with the guidelines of the Animal Care Committee of Dalian Medical University. Primary neuronal cultures were obtained from cerebral cortices of neonatal Sprague–Dawley rats (Laboratory Animal Center of Dalian Medical University) as described previously (Brewer 1997; Beaudoin et al. 2012). Briefly, tissue was digested in DMEM/F-12 (GIBCO, Invitrogen) with 2 mg/ml papain (Worthington) and 2 U/ml DNase I (Worthington) at 37 °C for 30 min and dissociated by mild trituration in DMEM/F12 containing 10 % FBS (GIBCO, Invitrogen) and 10 % donor equine serum (Hyclone). For survival analysis and immunocytochemistry, 5–7 × 10^5^/ml dissociated cells were plated in 24-well plates or coverslips previously coated with poly-D-lysine (PDL; Sigma). For biochemical procedures, 1–2 × 10^6^/ml cells were seeded in six-well plates coated with PDL. Cultures were maintained at 37 °C in a humidified atmosphere of 95 % O_2_ and 5 % CO_2_. After 4 h, mediums were replaced and cells were maintained with neurobasal medium (Invitrogen) supplemented with 2 % B-27 supplement (PAA Laboratories) and 0.5 mM glutamine. Half of the medium was refreshed every 3 days. All experimental treatments were performed in vitro on day 9 (DIV9). Immunofluorescence staining with anti-microtubule associated protein-2 (MAP-2; Sigma) antibody and anti-glial fibrillary acidic protein (GFAP; Millipore Chemicon) antibody revealed that cultures contained more than 90 % neurons.

#### Preparation of conditioned medium from AMSCs

AMSCs at passages 4–6 were seeded on 35 mm culture dishes with DMEM at a density of 10^5^/ml. After 48 h, the confluent cells were washed once with PBS and then incubated in neurobasal medium containing B-27 supplement. After another 18–20 h, the medium was collected and designated as conditioned medium (CM).

### Experimental treatments

Excitotoxic death was induced in DIV9 rat cortical neurons by exposure to different concentrations of glutamate (10, 50 and 100 μM) supplemented with 10 μM glycine (assist agonist of NMDA receptors) for 15 min at 37 °C. The medium was then replaced with either the original culture medium or different concentrations of AMSC-CM ranging from 10 to 100 % (v/v) and cells continued to be cultured for 18 h. Control groups were treated under the same conditions but in absence of glutamate and glycine.

### Co-culture of AMSCs and neurons

Following exposure to glutamate for 15 min, neurons were co-cultured with AMSCs. AMSCs were separated from neurons with a porous membrane (0.4 μm) which allowed for the exchange of molecules but prohibited cell-cell direct contact. Cortical neurons were cultured in 12- or 6-well plates for 9 days in vitro prior to excitotoxicity. AMSCs were plated in 12- or 6-well inserts (polyester, 1.12 cm^2^; Corning) and grown to 80–100 % confluence in serum-containing growth medium. Prior to placing AMSCs-containing inserts into plates containing neurons, AMSCs were rinsed twice with PBS and medium was changed to neuronal medium, consequently removing serum from the co-culture. Cells were co-cultured for 18 h and then neural damages were assessed.

### Assessments of cell damages

After cortical neurons were cultured in 24-well plates for 9 days, 100 μM glutamate and 10 μM glycine was added and cultured for 15 min at 37 °C. The medium was then replaced with either the original culture medium or the optimum concentration of CM, or inserts containing AMSCs were added to damaged neurons. After 18 h, cell damages were evaluated by trypan blue dye (Sigma) and lactate dehydrogenase (LDH) release. Trypan blue dye was performed as previously described with modification. 18 h after insult, culture medium was removed and replaced with 0.12 mg/ml trypan blue dissolved in PBS. Cells were incubated at room temperature for 10 min and washed with PBS. Cultures were then fixed with 4 % paraformaldehyde for 30 min and cells were visualized under a phase-contrast microscope. Total numbers of dead and intact neurons were counted and expressed as a percentage of the total number of cells. For statistical purposes, the average of ten neighbor fields for each condition was used as a single data. LDH was assayed using the Cytotoxicity Detection Kit ^plus^ (Roche Applied Science) according to the manufacturer’s instructions. Results were read on an EL808 microplate assay reader (Gene Company Limited). Untreated cells were used for control groups and the culture medium without cells was used as background control. Data represent the percentage relative to control treated with lysis buffer. Each group contained three samples and experiments were repeated at least three times.

### TUNEL and Nissl Staining

An In Situ Cell Death Detection Kit (Fluorescein, Roche Applied Science) based on the TUNEL assay was used to evaluate apoptotic cell death following the manufacturer’s instructions. For negative controls, reactions were performed by omitting TUNEL enzyme TdT and cells were incubated with the label solution provided in the kit. No reactivity was observed when TdT was absent. Cells were stained with fluorescent Nissl dye (TRITC, Molecular Probe) to label the total neurons and were observed with a fluorescence microscope (LEICA, DFC500). The number of TUNEL-positive cells and Nissl-positive cells were counted in at least three separate experiments per treatment condition without knowledge of treatment history.

### Immunofluorescent labeling of GAP-43

Briefly, cultured neurons were fixed with 4 % paraformaldehyde for 30 min and permeabilized with 0.3 % Triton X-100 for 20 min at room temperature. After blocking unspecific binding sites with 10 % bovine serum albumin (BSA; Sigma), cells were incubated overnight with monoclonal rabbit anti-GAP-43 (1:200; Abcam) antibody at 4 °C. Cells were then washed for three times in 0.01 M PBS and incubated with specific secondary antibody conjugated FITC (1:100; goat anti-rabbit; Sigma) for 60 min at room temperature. For negative controls, reactions were performed by omitting the primary antibody. No reactivity was observed when the primary antibody was absent. Nuclei were counterstained with Hoechst33258 (10 μg/ml; Sigma). Cells were observed and photographed with a fluorescence microscope (LEICA, DFC500).

### Western blotting analysis for detection of the protein levels of GAP-43

Protein levels of GAP-43 under different experiment conditions were detected by Western blot assay with β-actin as an internal control. Cultured neurons in six-well plates were washed three times with cold PBS and lysed on ice with RIPA buffer containing protease inhibitor for 30 min. Samples were centrifuged at 12,000 rpm for 20 min at 4 °C and the supernatant was collected for Western blot assays. Protein levels in the supernatant were determined using a BCA kit (Beyotime Biotechnology). 35 μg of protein from each sample was loaded on 12 % SDS-PAGE gels. After separation by electrophoresis, proteins were transferred to PVDF membranes (Millipore) and blocked unspecific binding with 5 % non-fat dry milk. Membranes were incubated with rabbit anti-GAP-43 monoclonal antibody (1:500; Abcam) overnight at 4 °C. Following three washes with TBST, the blots were incubated with goat anti-rabbit IgG-HRP (1:1,000) for 1 h at room temperature. Immunoreactive bands were visualized with an ECL detection kit (Thermo) on light sensitive film. Bands were analyzed with Gel-Pro Analyze4 software. Data represent the percentage relative to the control group.

### Measurement of ATP levels

Total intracellular ATP concentrations in neurons were measured with a luciferin-luciferase assay-based commercial kit (Bioassay). Briefly, neurons grown in 6-well plates were washed three times with cold PBS and then lysis buffer was added to each well. Cells were collected after thoroughly scraping the culture at the well bottom. Cell lysates were centrifuged at 12,000 rpm for 20 min. The supernatant was used for ATP assay. Luminescence was measured using a luminometer (Corning). A standard curve and equation were generated using an ATP standard and used to calculate ATP concentrations in samples.

### Measurement of NAD^+^ and NADH levels

Total intracellular NAD^+^/NADH concentrations in neurons were measured using an Enzychrom^TM^ NAD^+^/NADH assay kit (Bioassay). The assay is based on an enzyme-catalyzed kinetic reaction. Briefly, NAD^+^/NADH was extracted from cells at 60 °C for 5 min, neutralized with the opposite extraction and then centrifuged at 14,000 rpm for 5 min. NAD^+^ extracts in the supernatant were converted to NADH by enzymatic cycling with lactate dehydrogenase, which reduces MTT to formazan. Optical density was measured at 560 nm using a plate reader (Gene Company Limited). The intensity of the reduced produce color is proportional to the NAD^+^/NADH concentration in the sample. A standard curve and equation were generated using an NAD standard and used to calculate NAD^+^/NADH concentrations in samples.

### Determination of mitochondrial membrane potential

Mitochondrial membrane potential (MMP) was evaluated with the fluorescent probe tetramethylrhodamine ethyl ester (TMRE, Invitrogen) using methods described previously. Cells were loaded with 10nM TMRE at 37 °C for 30 min, washed, and maintained in culture medium throughout experiments. Images of TMRE fluorescence were captured with a phase-contrast fluorescent microscope (LEICA, DFC500) at excitation and emission wavelengths of 488 nm and 525 nm respectively. The mean intensity of fluorescence was measured and calculated using Image-Pro Plus 6.0 software.

### Statistical analysis

Statistical analysis was performed using SPSS 11.5. Datas are presented as mean±SEM. The difference between mean values was determined by one-way ANOVA. *P* values of less than 0.05 were considered statistically significant.

## Results

### AMSC-CM attenuated neuronal damages in a concentration-dependent manner

**Fig. 1:**
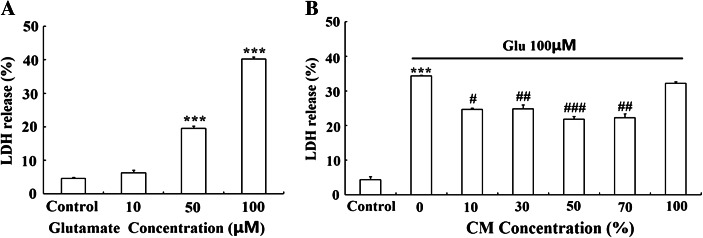
Glutamate was toxic to cultured cortical neurons and AMSC-CM reduced glutamate-induced neuronal damage in a concentration-dependent manner. **a** Cortical neurons at DIV9 were exposed to glutamate (10–100 μM) supplemented with 10 μM glycine for 15 min and the original culture medium was replaced. Cell damage was determined by LDH release after 18 h. Glutamate induced a dose-dependent neuronal damage. 100 μM glutamate supplemented with 10 μM glycine was chosen for all subsequent experiments. **b** Different concentrations of CM were added to damaged neurons following glutamate exposure and cell damage was evaluated by LDH release after 18 h. Note that glutamate-induced neuron injury was attenuated most effectively by 50 % CM, whereas 100 % CM did not mediate any neuroprotective effects. As the optimal concentration of CM, 50 % CM was used for all subsequent experiments. Data are presented as mean±SD. ^***^ *P* < 0.001 versus control group, ^#^ *P* < 0.05, ^##^ *P* < 0.01, ^###^ *P* < 0.001 versus glutamate treated group. One-Way ANOVA

To examine the glutamate excitotoxicity on neurons, DIV9 primary cultured cortical neurons were treated with glutamate (10–100 μM) supplemented with 10 μM glycine for 15 min and neurobasal medium was replaced and maintained for 18 h. Cell damage was measured by LDH release. The results showed that glutamate exposure displayed evident toxicity in cultured cortical neurons and led to cell damage in a dose-dependent manner (Fig. 1a). 50 μM glutamate induced a modest but significant LDH release (19.48 ± 0.77 %). More extensive damage occurred with 100 μM glutamate (40.26 ± 0.60 %). 100 μM glutamate supplemented with 10 μM glycine was used as an excitotoxicity model for subsequent experiments because of LDH release from nearly half of the cells at this dose.

In order to investigate whether AMSC-CM could protect neurons against glutamate excitotoxicity, different concentrations of CM (10–100 %) were added to damaged neurons caused by 15 min of exposure to glutamate. Cells were incubated for another 18 h. The neuroprotective effect of CM was evaluated by LDH release. As shown in Fig. 1b and Table 1, CM reduced glutamate-induced neuronal injury in a concentration- dependent manner with a maximum protective effect at 50 % CM. At CM contents lower than 50 % or at 70 % CM, protective effects were less pronounced. Note that 100 % CM (all medium replaced with CM) did not mediate any protection. 50 % CM was used for all subsequent experiments because of the optimal neuroprotection (lowest LDH release).

**Table 1:** AMSC-CM reduced glutamate-induced neuronal damage in a concentration-dependent manner

Group	Control	Glu	10 % CM	30 % CM	50 % CM	70 % CM	100 % CM
LDH release (%)	4.37 ± 0.89	34.34 ± 0.23	24.79 ± 0.33	24.67 ± 1.18	21.78 ± 0.86	22.29 ± 1.13	32.14 ± 0.54
*P* value		0.000^***^	0.022^#^	0.006^##^	0.000^###^	0.002^##^	0.733

Different concentrations of CM were added to damaged neurons and cell damage was evaluated by LDH release after 18 h. Data are presented as mean±SD. ^***^ *P* < 0.001 compared to control group, ^#^ *P* < 0.05, ^##^ *P* < 0.01, ^###^ *P* < 0.001 compared to glutamate treated group. One-Way ANOVA

### AMSCs and CM protected cortical neurons against glutamate-induced damages and apoptosis

**Fig. 2:**
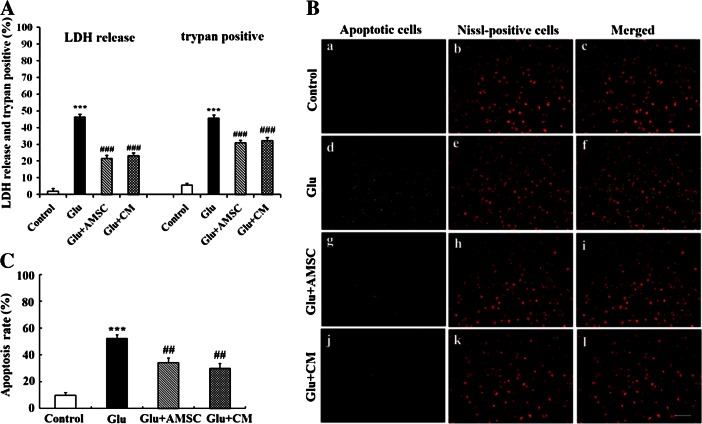
AMSCs reduced glutamate-induced damage and apoptosis in cortical neurons. Following 15 min of glutamate treatment, neurons were co-cultured with AMSCs or medium was replaced with AMSC-CM. Cell damages and apoptosis were determined by LDH release, trypan blue dye and TUNEL staining after 18 h. **a** Quantitative analysis of trypan blue dye and the result of LDH release. **b** Representative photomicrographs of total neurons stained with Nissl (*red*) and apoptotic nuclei stained with TUNEL (*green*) and merged photos were shown. (*a–c*) untreated control group; (*d–f*) neurons treated with glutamate alone; (*g–i*) damaged neurons co-cultured with AMSCs; (*j–l*) glutamate-treated neurons in the presence of AMSC-CM. **c** Quantitative analyses of Nissl and TUNEL co-staining. Data are presented as mean±SD. ^***^ *P* < 0.001 versus control group, ^#^ *P* < 0.05, ^##^ *P* < 0.01, ^###^ *P* < 0.001 versus glutamate treated group. One-Way ANOVA. Scar bar 200 μm

To investigate whether AMSCs could mediate neuroprotection, conditioned medium or a transwell co-culture system, in which cortical neurons were co-cultured in indirect contact with AMSCs and separated by a semiporous membrane (pore size 0.4 μm) allowing for the exchange of small molecules, were used. As shown in Fig. 2a, glutamate induced cortical neurons death as measured 18 h post-incubation by LDH release and trypan blue assay. However, when neurons were co-cultured with AMSCs or CM, LDH release and the number of trypan-positive cells significantly decreased compared with glutamate treated group. But comparing with uninjured controls, cell damage was only partially preserved, indicating a limitation of the protective effect of AMSCs on injured neurons. Furthermore, there was no significant difference in cell damage between neurons co-cultured with AMSCs and those incubated with CM (*P* > 0.05 in all cases).

**Fig. 3:**
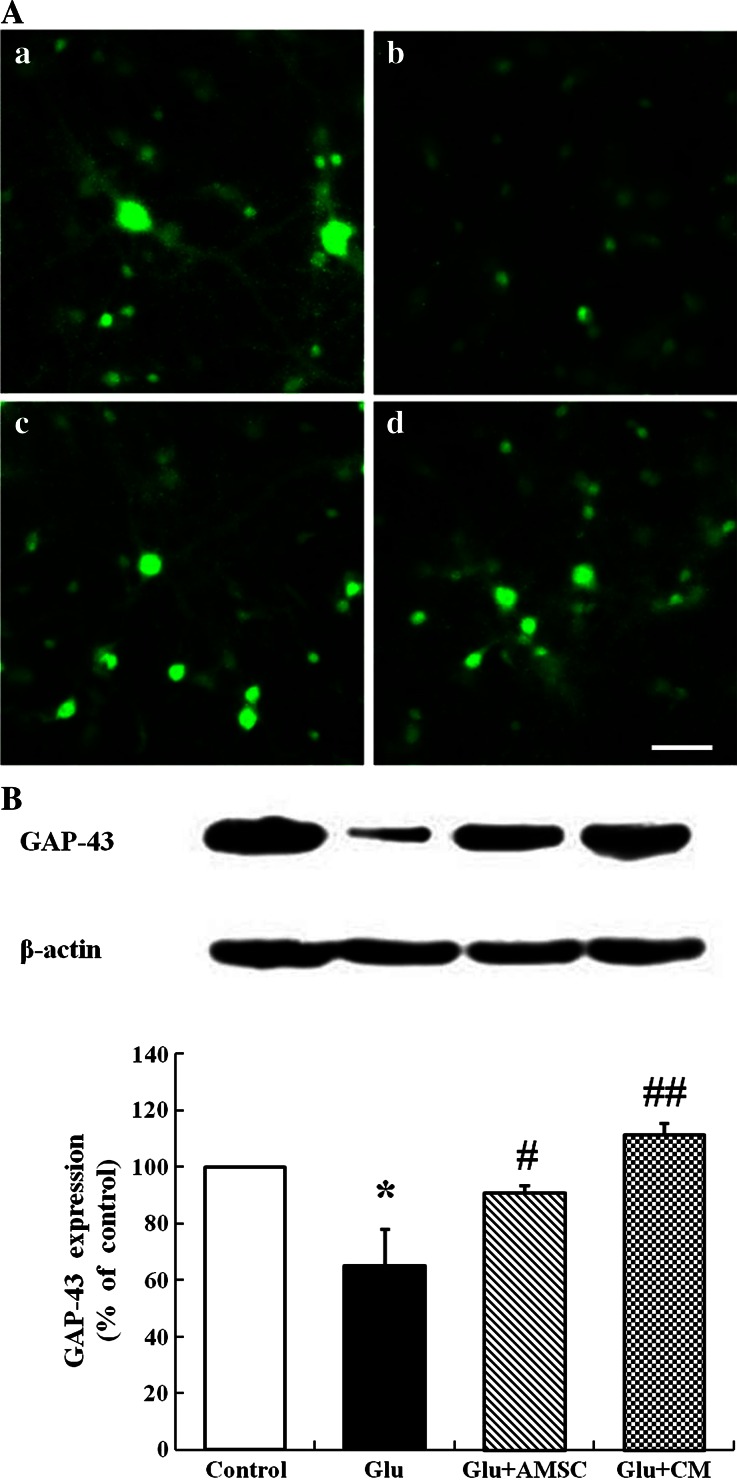
AMSCs restored GAP-43 expression and distribution in cortical neurons. After exposure to glutamate for 15 min, GAP-43 expression and distribution in neurons were measured by immunofluorescence labeling and Western blot. **a** Representative photomicrographs of GAP-43 (*a*) control group; (*b*) glutamate treated group; (*c*) AMSCs co-culture group; (*d*) AMSC-CM group. **b** GAP-43 levels were analyzed using Western blot. Protein loading was checked by stripping and re-probing the membrane with anti-actin antibody. Data are presented as mean±SD. ^*^ *P* < 0.05 versus control group, ^#^ *P* < 0.05, ^##^ *P* < 0.01 versus glutamate treated group. One-Way ANOVA. Scar bar 200 μm

To further explore whether AMSCs could reduce glutamate-induced neuron apoptosis, TUNEL and Nissl co-staining were performed. A low number of apoptotic cells was seen in the control group (9.80 ± 2.12 %, Fig. 2b [a–c]). The percentage of apoptotic cells increased dramatically in the glutamate group (52.36 ± 2.8 %, Fig. 2b [d–f]). However, apoptosis was suppressed in the AMSCs co-culture (34.32 ± 3.3 %, Fig. 2b [g–i]) and CM groups (30.08 ± 3.5 %, Fig. 2b [j–l]). The reduced percentage of apoptotic cells was still significantly higher (*P* < 0.05) than those in the uninjured control group (Fig. 3c). Similarly, no significant difference was seen in the percentage of apoptotic cells between neurons co-cultured with AMSCs and those incubated with CM.

### AMSCs and CM rescued GAP-43 expression and distribution in cortical neurons

To test the effects of glutamate on GAP-43 expression and distribution within cortical neurons, neurons were processed for GAP-43 immunofluorescent labeling and levels of GAP-43 were detected by Western blot. In the control group, GAP-43 was mainly distributed in neuronal bodies and processes. The fluorescence intensity of GAP-43 was homogeneously spread throughout the cytoplasm with weaker dotted distribution in the processes (Fig. 3a[a]). However, in glutamate treated group, GAP-43 intensity decreased markedly and GAP-43 was located neuronal bodies with little expression in processes (Fig. 3a[b]). AMSCs or CM caused an increase in GAP-43 fluorescence intensity and restored typical GAP-43 distribution in the presence of glutamate as compared with the glutamate-only treated group. The GAP-43 fluorescent signal was strong and the distribution in the cytoplasm remained homogeneous. The number of GAP-43-positive neurites increased in the co-culture and CM groups (Fig. 3a[c, d]). The results of Western blot analysis showed that glutamate reduced the level of GAP-43 protein compared with control group (Fig. 3b, 65.18 ± 12.96 %). GAP-43 expression increased markedly when damaged neurons were co-cultured with AMSCs (Fig. 3b, 91.08 ± 2.46 %) or CM (Fig. 3b, 111.62 ± 3.74 %). There was no significant difference (*P* > 0.05) in GAP-43 expression between the three experimental groups.

### AMSCs and CM prevented glutamate-induced reduction in ATP concentration

**Fig. 4:**
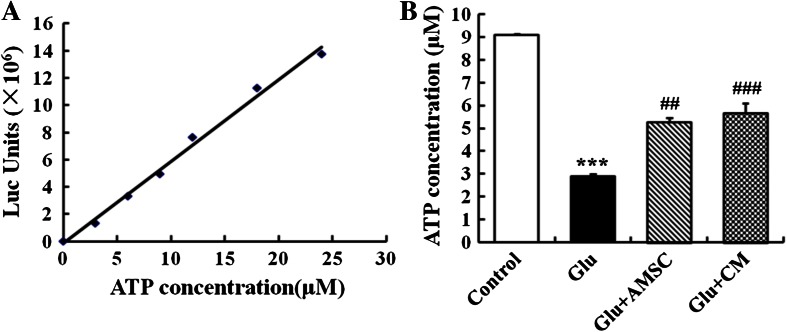
Glutamate caused a reduction in ATP concentration in cultured cortical neurons. AMSCs or CM prevented glutamate-induced ATP depletion. **a** A standard curve from an ATP assay showing a linear relationship between ATP levels and luminometer measurement values. **b** Total cellular ATP concentrations were evaluated with a luciferase-based ATP assay. Data are presented as mean±SD. ^***^ *P* < 0.001 compared to control group, ^##^ *P* < 0.01, ^###^ *P* < 0.001 compared to glutamate treated group. One-Way ANOVA

To examine the effect of glutamate on neuronal bioenergy homeostasis, a luciferase -based ATP assay was used to evaluate total ATP levels in cortical neurons. As a control, a standard curve showed a linear relationship between ATP concentration and fluorescence intensity (Fig. 4a). 100 μM glutamate induced a drastic and significant total ATP reduction (Fig. 4b, 9.07 ± 0.06 μM for control group, 2.89 ± 0.09 μM for glutamate group). AMSCs or CM restored partially the decrease in ATP levels after glutamate exposure (Fig. 4b, 5.28 ± 0.19 μM for co-culture group, 5.66 ± 0.42 μM for CM group).

### AMSCs and CM prevented NAD^+^ and NADH depletion and increased the ratio of NAD^+^/NADH

**Fig. 5:**
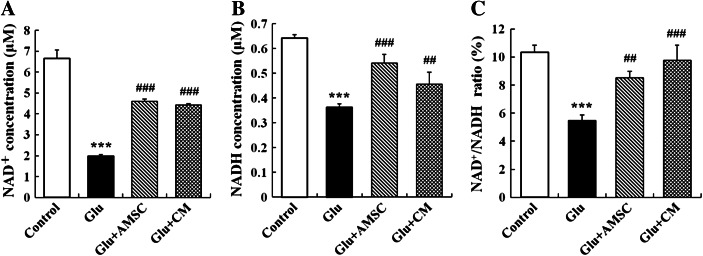
Glutamate resulted in a reduction in levels of NAD^+^ and NADH and a decrease in the ratio of NAD+/NADH in cortical neurons. AMSCs or CM prevented NAD^+^ and NADH depletion. **a** and **b** Total cellular NAD^+^ and NADH levels were measured with an enzymatic cycling assay. **c** Total cellular ratio of NAD^+^/NADH. Data are presented as mean±SD. ^***^ *P* < 0.001 compared to control group,^##^ *P* < 0.01,^###^ *P* < 0.001 compared to glutamate treated group. One-Way ANOVA

Following excitotoxic insult, total cellular NAD^+^ and NADH levels were measured using an enzymatic cyclic assay. When cortical neurons were exposed to glutamate (100 μM) in combination with glycine (10 μM), a significant decrease of total cellular NAD^+^ (Fig. 5a, 6.65 ± 0.41 μM for control group, 1.99 ± 0.07 μM for glutamate group) and NADH levels (Fig. 5b, 0.64 ± 0.01 μM for control group, 0.36 ± 0.01 μM for glutamate group) occurred within 18 h. Noticed that ratio of NAD^+^/NADH was also reduced following glutamate exposure (Fig. 5c, 10.36 ± 0.51 % for control group, 5.48 ± 0.39 % for glutamate group). When neurons were co-cultured with AMSCs or incubated with CM for 18 h, cellular NAD^+^ and NADH levels sharply increased to 4.60 ± 0.12 μM and 0.54 ± 0.04 μM in the co-culture group, respectively, and 4.42 ± 0.06 μM and 0.46 ± 0.05 μM in the CM group, respectively. The ratio of NAD^+^/NADH rose to 8.51 ± 0.48 % or 9.70 ± 1.09 % compared to the glutamate treated group.

### AMSCs and CM reversed glutamate-induced MMP collapse

**Fig. 6:**
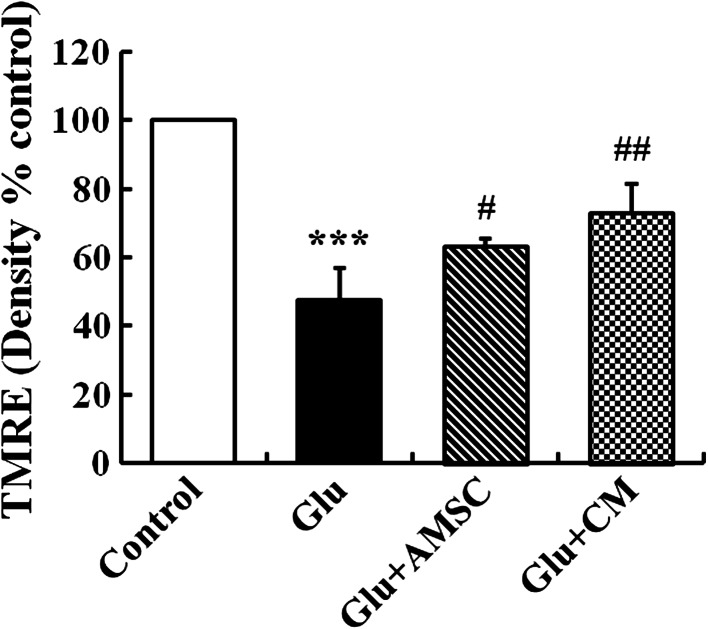
Glutamate reduced MMP in cultured cortical neurons. AMSCs or CM preserved MMP collapse following glutamate treatment. MMP was observed using a TMRE fluorescent probe. Image Pro-Plus 6.0 software was used for quantitative TMRE fluorescent density analysis. Data are presented as mean ± SD. ^***^ *P* < 0.001 compared to control group, ^#^ *P* < 0.05, ^##^ *P* < 0.01 compared to glutamate treated group. One-Way ANOVA

To test the impact of glutamate on mitochondrial function, changes in MMP in cultured cortical neurons were estimated using the fluorescent cationic dye TMRE, which preferentially labels active mitochondria based on the highly negative MMP. As shown in Fig. 6, glutamate caused a sharp reduction in active mitochondria (47.74 ± 8.42 % for control group). However, AMSCs or CM treatment resulted in a relative increase in active mitochondria compared to the glutamate treated group (73.0 ± 7.46 % and 63.2 ± 2.33 %, respectively).

## Discussion

Glutamate excitotoxicity is caused by a massive influx of calcium into neurons due to overstimulation of glutamate receptors and is closely related to neuronal cell injury in stroke, trauma and other chronic neurodegenerative diseases (Lipton and Rosenberg 1994; Lau and Tymianski 2010). In the present study, an in vitro model of glutamate excitotoxicity was used to investigate the neuroprotective effects of MSCs-CM on cortical neurons. The results indicate that AMSCs-CM are able to protect cortical neurons against glutamate excitotoxicity and give the first evidence that AMSCs-CM act by regulating GAP-43 expression and rescuing energy depletion and mitochondrial function.

Our understanding of how the nervous system repair occurs through somatic stem cells, particularly MSCs, has recently changed. MSCs appear to exert their therapeutic effects not because of their intrinsic ‘stemness’, but due to their capacity to release therapeutic molecules that can interact with the host environment (Prockop 2007). Indeed, studies of the MSCs secretome using high performance liquid chromatography-mass spectrometry (HPLC-MS) and antibody-related technologies have demonstrated that MSCs can secrete many kinds of neuro-regulatory molecules, cytokines, growth factors and chemokines which show neuroprotecive and neurorestorative effects, including increasing neuronal viability, inducing the proliferation and differentiation of endogenous neural stem/progenitor cells and promoting regeneration of nerve fibers at sites of injured (Salgado et al. 2009; Singer and Caplan 2011; Skalnikova et al. 2011). The co-culture system used in the present study allowed for the separation of AMSCs from neurons with a semiporous membrane, which prevented direct cell-cell contact and showed that trophic support plays an important role in protecting damaged neurons. In order to further evident the effects of trophic support, AMSC-CM was used for its ability to reduce neural cells injury. The findings reported here show that the neuroprotective effects of AMSC co-culture and CM are not significantly different and both prevent neuronal injury induced by glutamate excitotoxicity in the same extent. This suggests that the observed beneficial effects of AMSCs are mediated by soluble factors secreted by these AMSCs in their conditioned medium. Previous research supports this conclusion. Lu et al. found that MSCs protected PC12 cells from glutamate-induced apoptosis by secreting VEGF, HGF, BDNF and NGF, and neutralization antibody of these factors weakened the protective function of MSCs (Lu et al. 2011). Furthermore, in a recent paper Voulgari-Kokota et al. removed cell-derived microparticles from freshly-prepared MSC-CM using a step-wise centrifugation protocol and found that microvesicle-depleted CM had similar neuroprotective effects against NMDA as complete CM in cortical neurons (Voulgari-Kokota et al. 2012). These finding further indicates that soluble factors secreted by MSCs are what provide neuroprotection against neuronal injury. This is consistent with the view that effects of MSCs are not dependent on their fully integration into the damaged organs after transplantation.

One potential advantage of cell-based therapy to treat neurological disorders is the ability of transplanted cells to sense environmental factors related to tissue damages and response to the injured organs. Previous study has shown that trophic factors released by MSCs increase when they are co-cultured with the injured brain tissue extract (Chen et al. 2002). Isele et al. showed that the protective effects of MSCs on rat neurons against staurosporine (STS) or amyloid-beta peptide-induced apoptosis increased significantly if MSCs were first exposed to neurobasal medium from apoptotic neurons (Isele et al. 2007). But in the present study, remarkable differences in neuron damages were not found between AMSCs co-culture and CM treatment, indicating that any cross-talking between AMSCs and damaged neurons was not detectable under this system. The impact of an injured environment on the ability of MSCs to repair neural cells requires further investigation.

It is important to note that AMSC-CM reduced neural injury induced by glutamate excitotoxicity in a concentration-dependent manner, with an optimal protective effect at 50 % CM. At CM concentrations lower than 50 % or 70 %, the protective effect was diminished. Pure CM (100 %), however, did not lend any neuroprotection. Similarly, Isele NB et al. showed that 100 % BMSC-CM appeared to accelerate STS-mediated apoptosis (Isele et al. 2007). Other researchers have shown that AMSCs can release chemokine and pro-inflammatory factors that are detrimental to damaged tissues (Kilroy et al. 2007; Skalnikova et al. 2011). Adverse effects from MSCs may hasten neuronal death and increase tissue lesions, particularly when MSCs accumulate at the site of lesions. Recent observations demonstrated that intra-cerebroventricular transplantation of MSCs in mice with severe experimental autoimmune encephalomyelitis (EAE), an experimental model of multiple sclerosis, can induce the formation of cellular masses with collagen/fibronectin deposition in the brain parenchyma (Grigoriadis et al. 2011). Previous studies also indicated that MSCs migrated to and accumulated in brain lesion areas after systemically administered MSCs (Chen et al. 2001). However, Liu et al. had reviewed exosomes, a kind of microparticles secreted from cells and without immunogenicity to targeted cells, might be a potential therapeutic vector for metabolic brain diseases (Liu et al. 2013). Thus, microparticles from the AMSC-CM might mediate neuroprotective effects, reduce the toxic substance of conditioned medium and thus avoid the risk of stem cells differentiation into neoplasms.

The foremost regenerative targets following the CNS injury include increasing cell viability, promoting regeneration of axon and myelin and reorganizing synapses. GAP-43 is an intracellular growth-associated protein which appears to assist neuronal path-finding and branching during development and regeneration (Benowitz and Routtenberg 1997). In adults, GAP-43 induces neurotransmitter release, endocytosis and synaptic vesicle recycling by changing the presynaptic membrane. It is involved in neuronal differentiation, plasticity, axonal growth and regeneration and synaptic reconstruction after nerve injury. In vivo and in vitro experiments have demonstrated that GAP-43 plays an important role in the processes of regeneration following central and peripheral nervous system damage. Increased expression of GAP-43 in lesion areas after brain ischemia or trauma was related to the activation of endogenous repair mechanism and the promotion of axonal regeneration in the injured nervous system (Gianola and Rossi 2004). Additionally, GAP-43 was shown to trigger a significant increase in the regeneration of dorsal root ganglion (DRG) axons in adult mice after spinal cord injury in vivo (Bareyre et al. 2002). In the present study, the expression and distribution of GAP-43 after glutamate exposure and the effect of AMSCs on axonal regeneration was observed. The result showed the expression of GAP-43 and the number of GAP-43 positive neurites decreased significantly following glutamate treatment. These indicators increased dramatically to the normal levels when injured neurons were treated with AMSCs or CM, suggesting that AMSCs are able to promote axonal regeneration or outgrowth. The mechanisms may be correlated with neurotrophins or neuro-regulatory factors released by AMSCs which act as positive guidance molecules for axonal growth cone, inducing neurites sprouting and growth following injury. Previous work demonstrated that AMSCs transplantation induced peripheral nerve repair and activated nerve sprout growth in vivo and this ability of AMSCs depended on BDNF secretion (Lopatina et al. 2011). Earlier data indicated that activation of NMDA receptors suppressed GAP-43 expression and axonal outgrowth in hippocampal slice cultures and IGF-1 elevated GAP-43 expression (McKinney et al. 1999). Recently (Li et al. 2013) and (Liu et al. 2012). demonstrated that growth factors neuregulin-1β and IGF-1 could partially reverse decreased GAP-43 expression induced by glutamate in DRG neurons and these effects were involved in activation of PI3K/Akt and ERK1/2 signaling pathways. These reports suggest that AMSCs are able to secrete some soluble factors that activate endogenous restorative and survival mechanisms, increase GAP-43 expression and promote axonal regeneration after nerve injury.

Another important aspect of the present study is that AMSC-CM can reverse the energy depletion induced by glutamate exitotoxicity, as indicated by enhanced MMP, increased ATP, NAD^+^ and NADH concentrations, and elevated ratio of NAD^+^/NADH. Increasing evidences suggest that mitochondrial dysfunction play a vital role in glutamate excitotoxicity (Nicholls 2004; Nicholls et al. 2007). Recently, mitochondria have been recognized as the key organelle determining the fate of cells because of their central functions such as ATP synthesis, Ca^2+^ accumulation, superoxide generation and detoxification and storage of pro-apoptotic proteins (Orrenius 2004; Smaili et al. 2011). MMP is the key parameter controlling these mitochondrial functions, which are of great relevance to neuronal survival. When glutamate-induced elevation of cytoplasmic free calcium ([Ca^2+^]_c_) exceeds the mitochondrial Ca^2+^ set-point at which the organelle behaves as a buffer of [Ca^2+^]_c_, mitochondrial Ca^2+^ overloading occurs (Kiedrowski 1999). Consequently, it leads to decline of MMP and the opening of permeability transition pore (PTP) which disrupts the proton gradient across the inner mitochondrial membrane and causes the bioenergetic collapse and cell death. Several studies indicate that glutamate decreases the ATP concentration in neurons (De Cristobal et al. 2002; Parihar et al. 2008; Foo et al. 2012). The reason for this may be falling of ATP production or increasing of ATP consumption or both. It has been shown that glutamate continues to reduce ATP concentrations even after mitochondrial oxidative phosphorylation and ATP genesis is blocked, indicating that ATP depletion caused by glutamate is associated with increased ATP consumption (Foo et al. 2012). Other data showed a decrease in ATP production following glutamate excitotoxicity (Parihar et al. 2008). Glutamate reduces MMP contributing to a loss of the proton gradient across the mitochondrial membrane, uncoupling of the electron transport chain, inhibition of oxidative phosphorylation and reduction of ATP production. During excitotoxic cell injury, impaired oxidative phosphorylation results in the release of NAD^+^ from mitochondria, which is finally cleaved by glycohydrolases (Belenky et al. 2007; Soane et al. 2007). It is widely accepted that NAD^+^ is not only an important energy substrate and cofactor involved in many metabolic reactions (Bieganowski and Brenner 2004; Belenky et al. 2007), but acts as an adenosine donor and source of high energy phosphate for ATP synthesis (Sheline et al. 2000). NAD^+^ depletion inhibits glycolysis, the tricarboxylic acid (TCA) cycle and mitochondrial oxidative phosphorylation, all of which reduce ATP levels and lead to energy depletion in neurons (Sheline et al. 2000). Therefore, neuronal NAD^+^ bioenergetic state and NAD^+^/NADH redox state are pivotal factors for cell survival after excitotoxic insults (Liu et al. 2008, 2009).

Growth factors such as bFGF, BDNF and taurine have been shown to protect cerebellar granule neurons against excitotoxicity by increasing the glucose uptake and stabilizing the cytoplasmic calcium and mitochondrial electrochemical activity (El Idrissi and Trenkner 1999). A recent study reported that BMSCs genetically modified by Akt elevated ratios of phosphocreatine/ATP and increased phosphocreatine levels in surviving myocardial cells (Gnecchi et al. 2009). More recent data shows that MSC-mediated protection against glutamate excitotoxicity involves the reduction of glutamatergic signaling (Voulgari-Kokota et al. 2012). Changes in gene expression from an activity-dependent mature neuron to a more immature one that is associated with survival and regeneration plasticity, may be a cause of neuronal desensitization to glutamate. Fu H et al. by analyzing neuronal mitochondrial proteomics found that glutamate altered the expression patterns of mitochondrial proteins involved in energy metabolism (electron transport chain, TCA cycle and glucose metabolism), oxidative stress and apoptosis (Fu et al. 2007). Tacrine reversed the expression pattern of these proteins, as well as the decline in MMP, ATP production and neuronal cell death following glutamate treatment. Based on these results and those of previous studies, we surmise that the protective effect of AMSCs against glutamate excitotoxicity observed in the present study might be due to AMSCs themselves or bioactive molecules secreted by them changing the gene expression associated with energy metabolism in neurons, maintaining normal mitochondrial functions and regulating mitochondrial-associated apoptotic signaling pathways. However, the exact mechanisms of MSC-mediated benefit in energy depletion during excitotoxicity need further be explored.

In conclusion, we report that AMSC-CM is able to protect cortical neurons against damage and apoptosis caused by glutamate excitotoxicity, ameliorate a decrease in both GAP-43 expression and the number of GAP-43 positive neurites and prevent glutamate-induced energy depletion. The neuroprotective effects of AMSCs may be related to the release of soluble factors. These findings may provide a new stem cell application method for the treatment of nervous system diseases. But a detailed understanding of the mechanisms of MSC-mediated benefits within the CNS and the identification of the soluble factors mediating MSCs protection will be necessary for further development of the clinical application of MSC-based therapy for neurological disorders.

## Abbreviations

CNS: Central nervous system
NMDA: N-Methyl-D-aspartic acid
GAP-43: Growth associated protein-43
MSCs: Mesenchymal stem cells
AMSCs: Adipose-derived mesenchymal stem cells
CM: Conditioned medium
BDNF: Brain derived neurotrophic factor
bFGF: Basic fibroblast growth factor
IGF-1: Insulin-like growth factor
VEGF: Vascular endothelial growth factor
HGF: Hepatocyte growth factor
NGF: Nerve growth factor
DMEM: Dulbecco’s modified Eagle medium
FBS: Fetal bovine serum
EDTA: Ethylenediaminetetraacetic acid
PDL: Poly-D-lysine
PBS: Phosphate-buffered saline
LDH: Lactate dehydrogenase
TUNEL: TdT-mediated dUTP nick-end labeling
PVDF: Polyvinylidene fluoride
SDS: Sodium dodecyl sulfate
NAD^+^: Nicotinamide adenosine denucleotide
NADH: Nicotinamide adenine denucleotide hydrogen
TMRE: Tetramethylrhodamine ethyl ester
ATP: Adenosine triphosphate
MMP: Mitochondrial membrane potential
PTP: Permeability transition pore
TCA: Tricarboxylic acid
